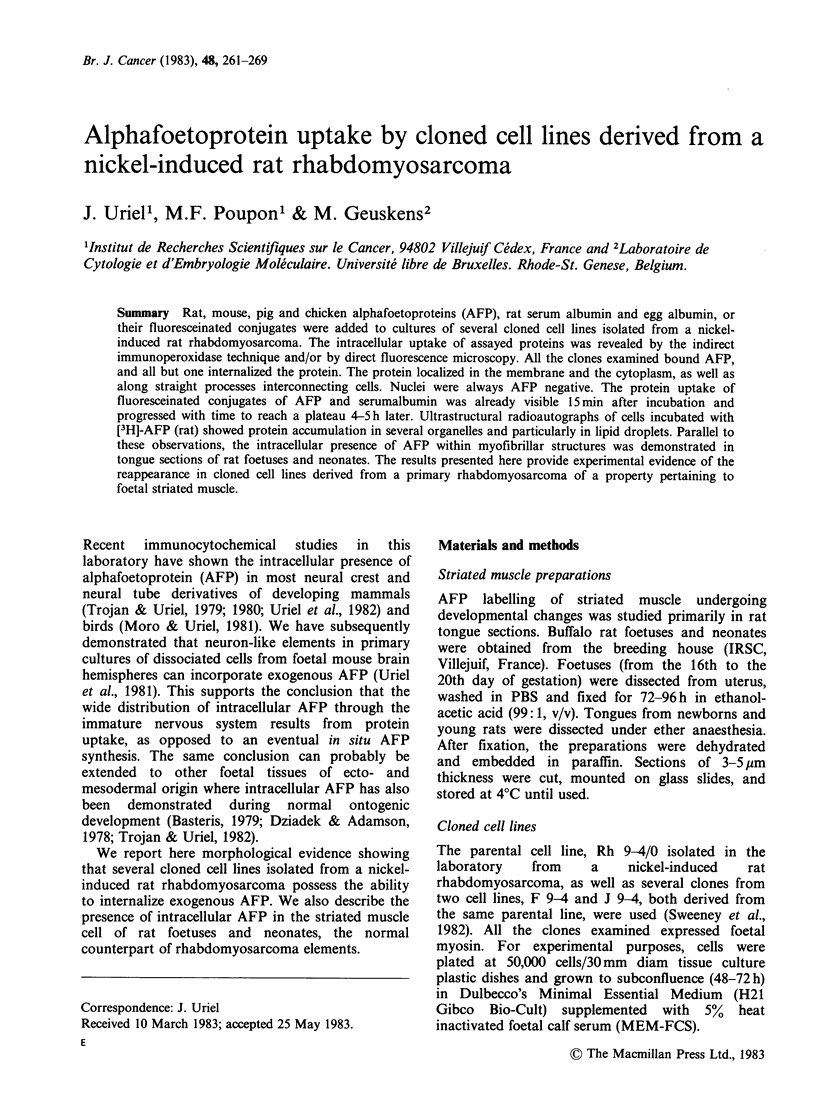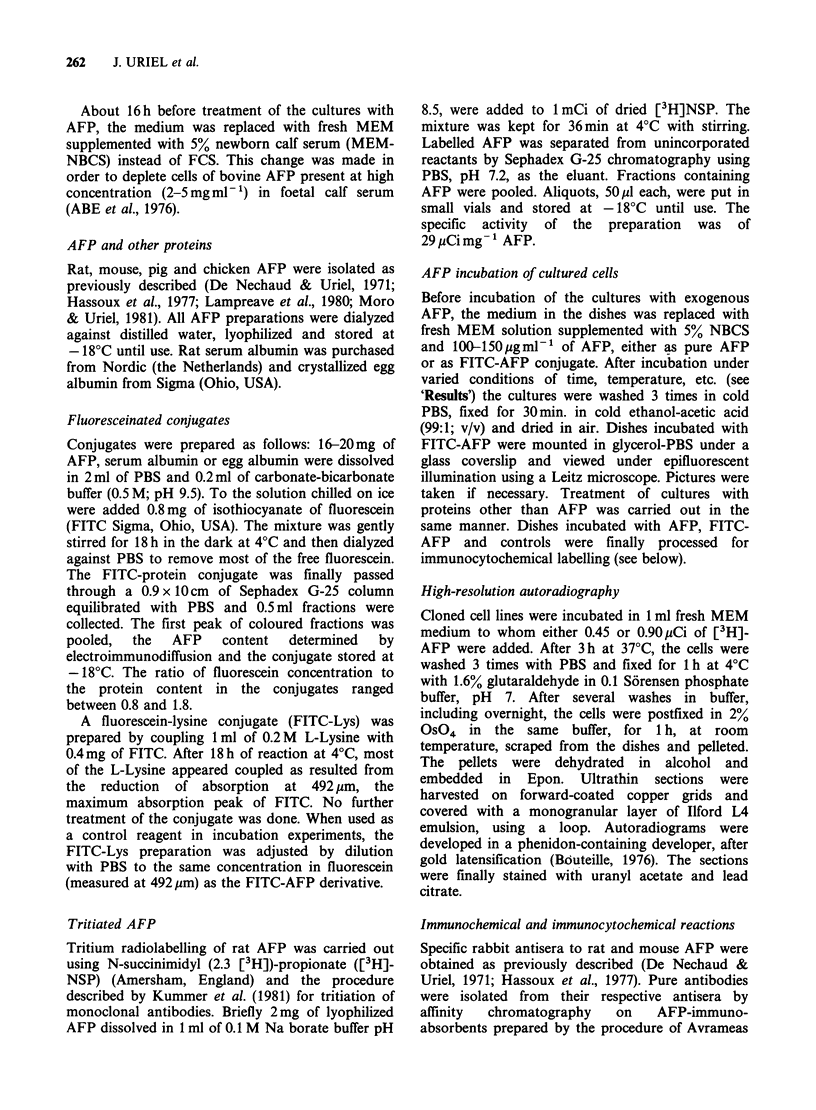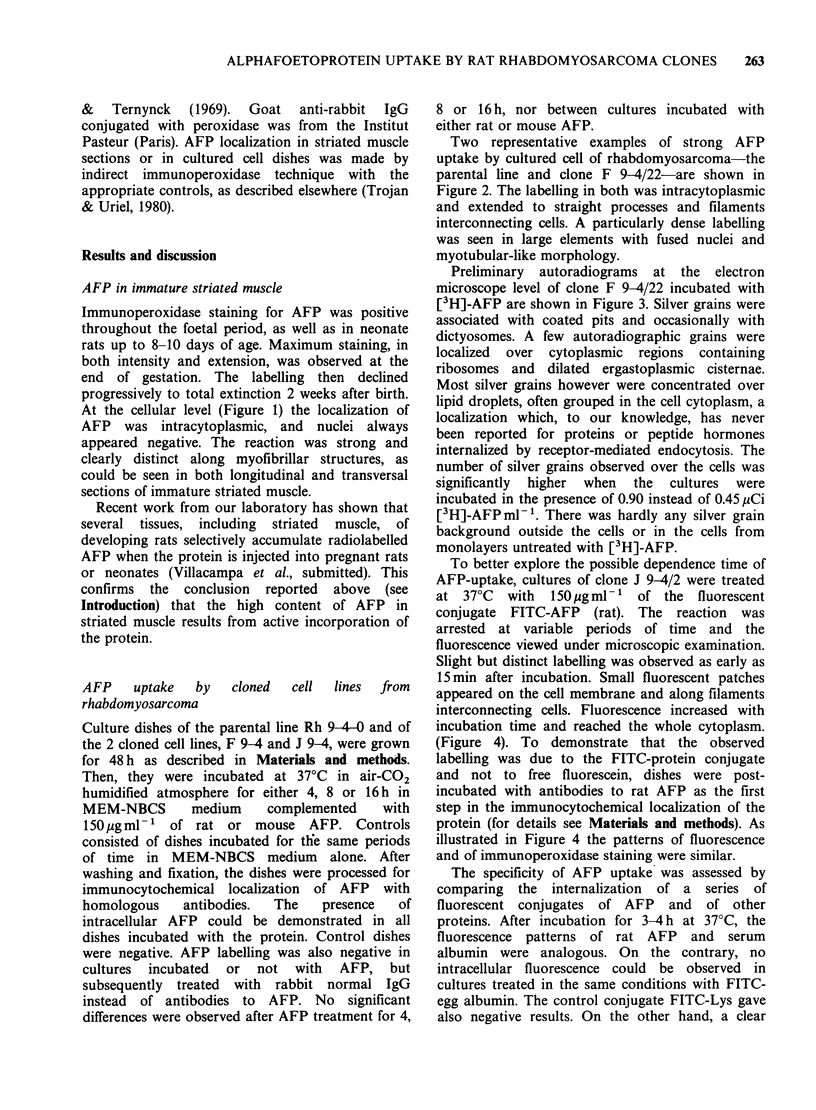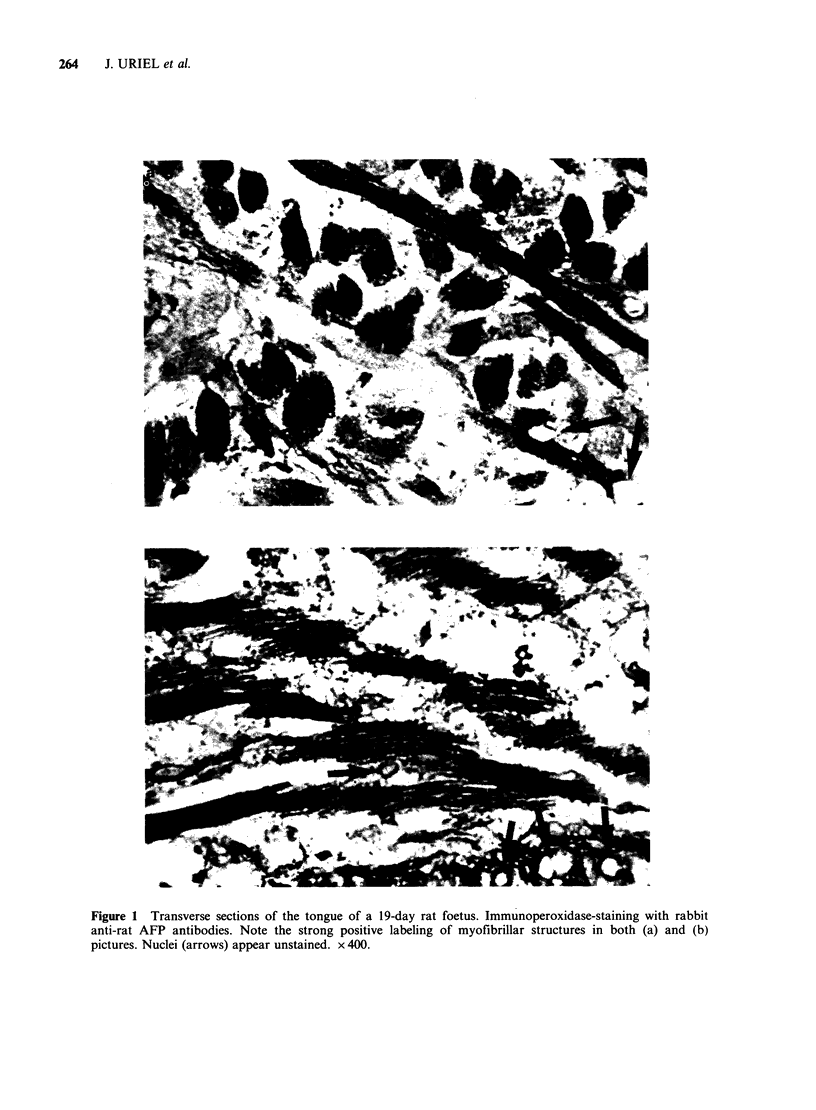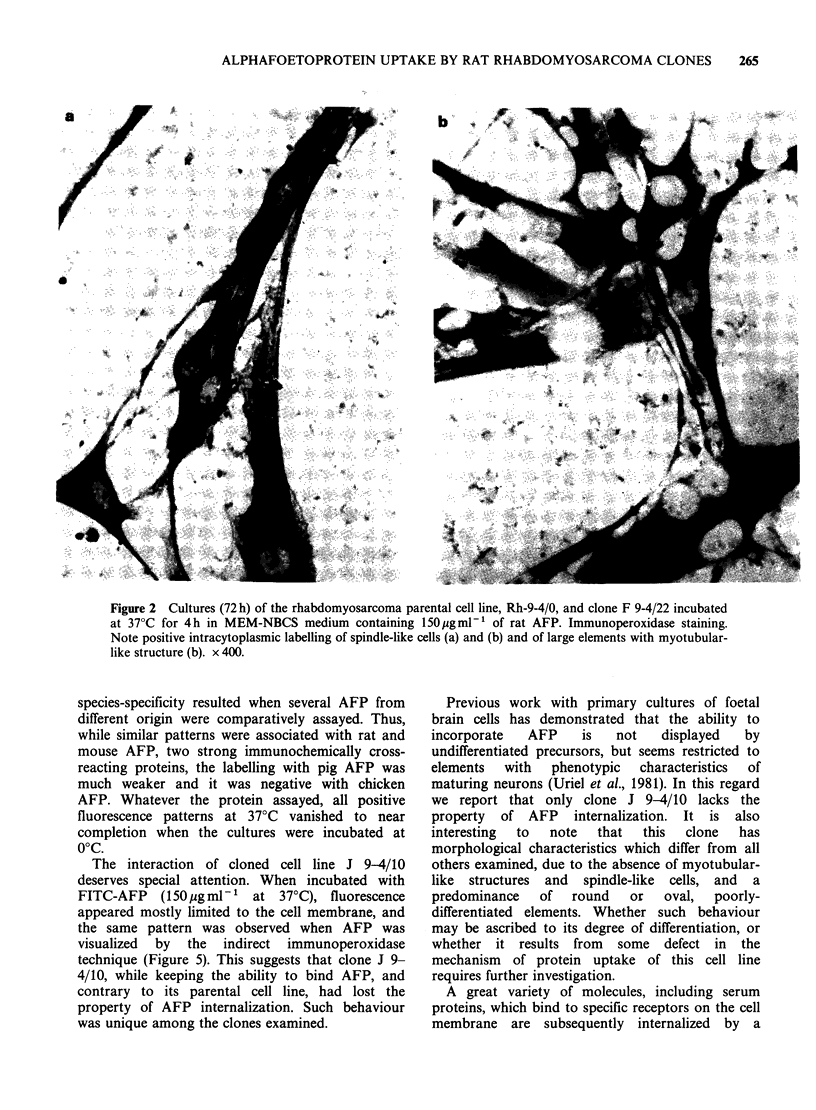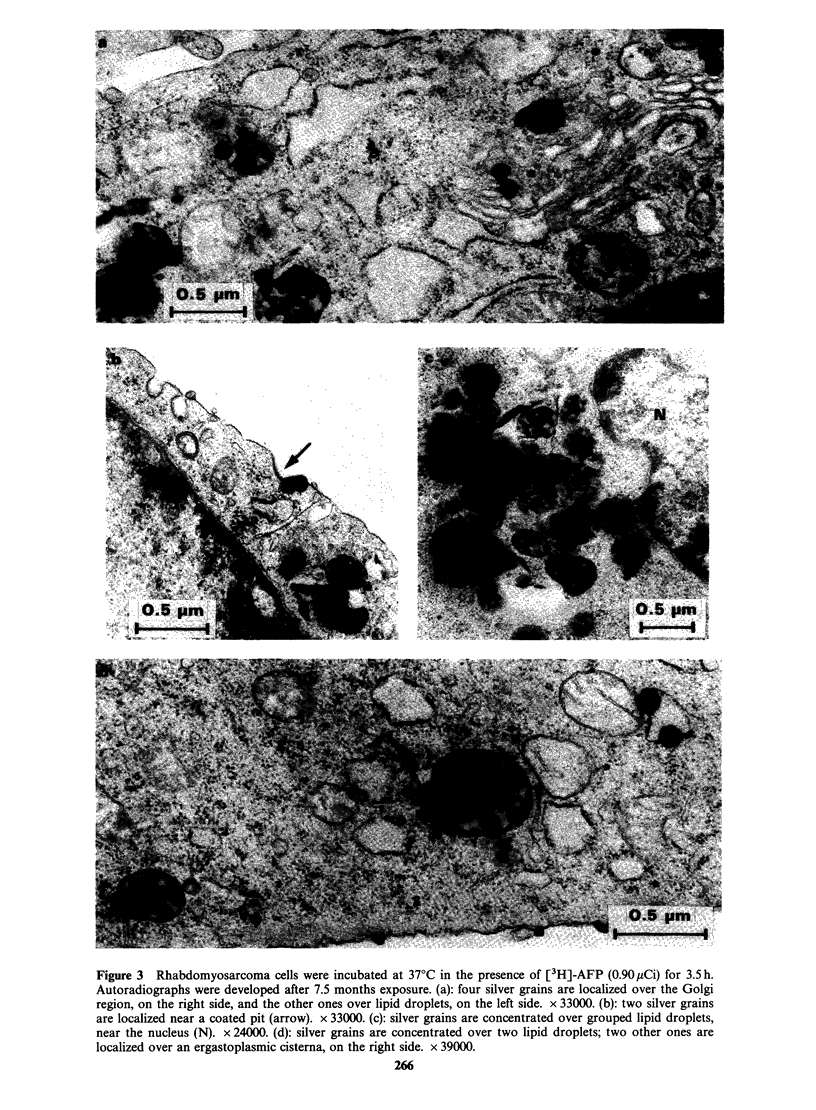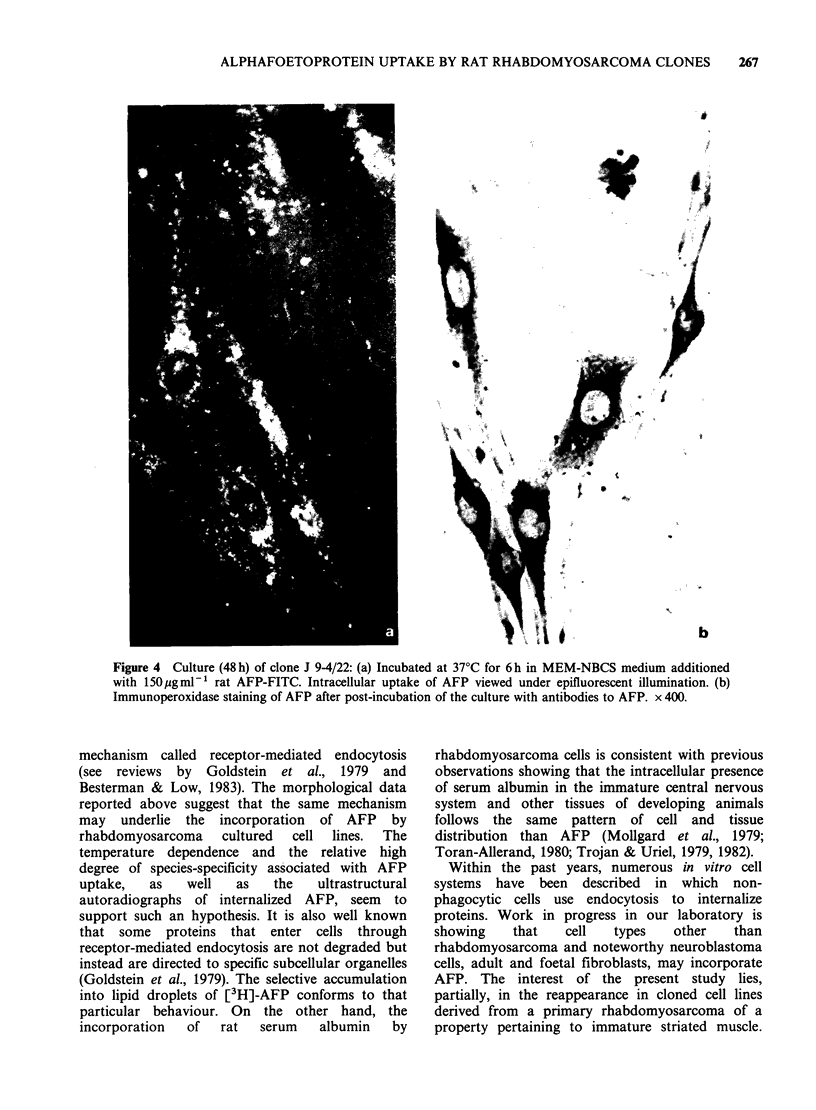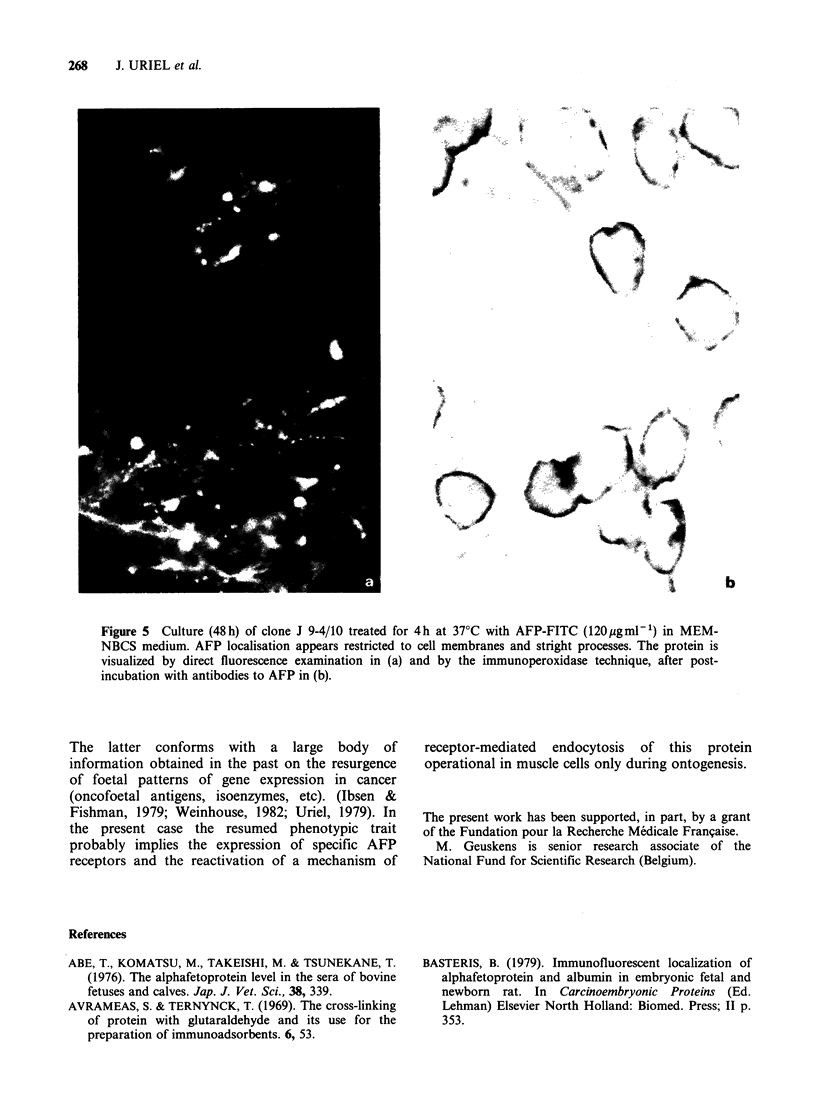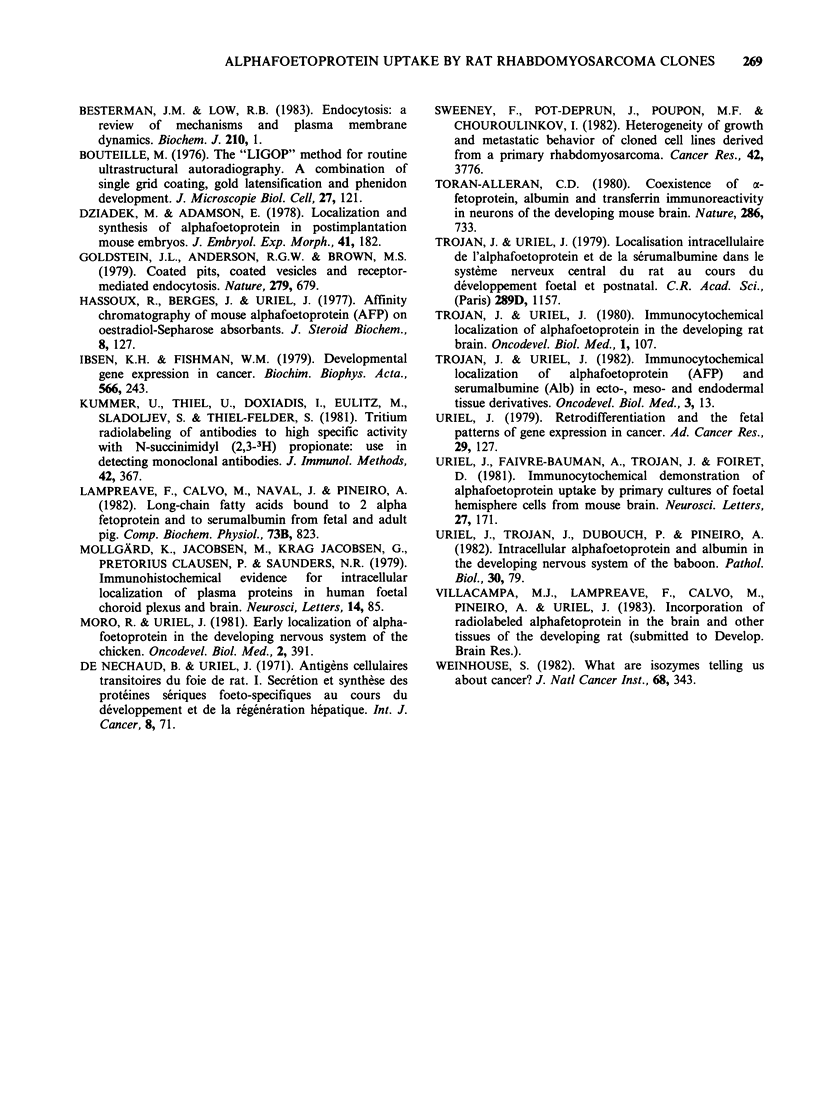# Alphafoetoprotein uptake by cloned cell lines derived from a nickel-induced rat rhabdomyosarcoma.

**DOI:** 10.1038/bjc.1983.181

**Published:** 1983-08

**Authors:** J. Uriel, M. F. Poupon, M. Geuskens

## Abstract

**Images:**


*Br. J. Cancer* (1983), **48**, 261–269

### Alphafoetoprotein uptake by cloned cell lines derived from a nickel-induced rat rhabdomyosarcoma

J. Uriel[1], M.F. Poupon[1] & M. Geuskens[2]

[1]*Institut de Recherches Scientifiques sur le Cancer, 94802 Villejuif Cédex, France and* [2]*Laboratoire de Cytologie et d'Embryologie Moléculaire. Université libre de Bruxelles. Rhode-St. Genese, Belgium.*

**Summary** Rat, mouse, pig and chicken alphafoetoproteins (AFP), rat serum albumin and egg albumin, or their fluoresceinated conjugates were added to cultures of several cloned cell lines isolated from a nickel-induced rat rhabdomyosarcoma. The intracellular uptake of assayed proteins was revealed by the indirect immunoperoxidase technique and/or by direct fluorescence microscopy. All the clones examined bound AFP, and all but one internalized the protein. The protein localized in the membrane and the cytoplasm, as well as along straight processes interconnecting cells. Nuclei were always AFP negative. The protein uptake of fluoresceinated conjugates of AFP and serumalbumin was already visible 15 min after incubation and progressed with time to reach a plateau 4–5 h later. Ultrastructural radioautographs of cells incubated with [$^3$H]-AFP (rat) showed protein accumulation in several organelles and particularly in lipid droplets. Parallel to these observations, the intracellular presence of AFP within myofibrillar structures was demonstrated in tongue sections of rat foetuses and neonates. The results presented here provide experimental evidence of the reappearance in cloned cell lines derived from a primary rhabdomyosarcoma of a property pertaining to foetal striated muscle.

Recent immunocytochemical studies in this laboratory have shown the intracellular presence of alphafoetoprotein (AFP) in most neural crest and neural tube derivatives of developing mammals (Trojan & Uriel, 1979; 1980; Uriel *et al.*, 1982) and birds (Moro & Uriel, 1981). We have subsequently demonstrated that neuron-like elements in primary cultures of dissociated cells from foetal mouse brain hemispheres can incorporate exogenous AFP (Uriel *et al.*, 1981). This supports the conclusion that the wide distribution of intracellular AFP through the immature nervous system results from protein uptake, as opposed to an eventual *in situ* AFP synthesis. The same conclusion can probably be extended to other foetal tissues of ecto- and mesodermal origin where intracellular AFP has also been demonstrated during normal ontogenic development (Basteris, 1979; Dziadek & Adamson, 1978; Trojan & Uriel, 1982).

We report here morphological evidence showing that several cloned cell lines isolated from a nickel-induced rat rhabdomyosarcoma possess the ability to internalize exogenous AFP. We also describe the presence of intracellular AFP in the striated muscle cell of rat foetuses and neonates, the normal counterpart of rhabdomyosarcoma elements.

Correspondence: J. Uriel
Received 10 March 1983; accepted 25 May 1983.

### Materials and methods

### *Striated muscle preparations*

AFP labelling of striated muscle undergoing developmental changes was studied primarily in rat tongue sections. Buffalo rat foetuses and neonates were obtained from the breeding house (IRSC, Villejuif, France). Foetuses (from the 16th to the 20th day of gestation) were dissected from uterus, washed in PBS and fixed for 72–96 h in ethanol-acetic acid (99:1, v/v). Tongues from newborns and young rats were dissected under ether anaesthesia. After fixation, the preparations were dehydrated and embedded in paraffin. Sections of 3–5 $\mu$m thickness were cut, mounted on glass slides, and stored at 4°C until used.

### *Cloned cell lines*

The parental cell line, Rh 9–4/0 isolated in the laboratory from a nickel-induced rat rhabdomyosarcoma, as well as several clones from two cell lines, F 9–4 and J 9–4, both derived from the same parental line, were used (Sweeney *et al.*, 1982). All the clones examined expressed foetal myosin. For experimental purposes, cells were plated at 50,000 cells/30 mm diam tissue culture plastic dishes and grown to subconfluence (48–72 h) in Dulbecco's Minimal Essential Medium (H21 Gibco Bio-Cult) supplemented with 5% heat inactivated foetal calf serum (MEM-FCS).

© The Macmillan Press Ltd., 1983

About 16 h before treatment of the cultures with AFP, the medium was replaced with fresh MEM supplemented with 5% newborn calf serum (MEM-NBCS) instead of FCS. This change was made in order to deplete cells of bovine AFP present at high concentration (2–5 mg ml$^{-1}$) in foetal calf serum (ABE *et al.*, 1976).

*AFP and other proteins*

Rat, mouse, pig and chicken AFP were isolated as previously described (De Nechaud & Uriel, 1971; Hassoux *et al.*, 1977; Lampreave *et al.*, 1980; Moro & Uriel, 1981). All AFP preparations were dialyzed against distilled water, lyophilized and stored at −18°C until use. Rat serum albumin was purchased from Nordic (the Netherlands) and crystallized egg albumin from Sigma (Ohio, USA).

*Fluoresceinated conjugates*

Conjugates were prepared as follows: 16–20 mg of AFP, serum albumin or egg albumin were dissolved in 2 ml of PBS and 0.2 ml of carbonate-bicarbonate buffer (0.5 M; pH 9.5). To the solution chilled on ice were added 0.8 mg of isothiocyanate of fluorescein (FITC Sigma, Ohio, USA). The mixture was gently stirred for 18 h in the dark at 4°C and then dialyzed against PBS to remove most of the free fluorescein. The FITC-protein conjugate was finally passed through a 0.9 × 10 cm of Sephadex G-25 column equilibrated with PBS and 0.5 ml fractions were collected. The first peak of coloured fractions was pooled, the AFP content determined by electroimmunodiffusion and the conjugate stored at −18°C. The ratio of fluorescein concentration to the protein content in the conjugates ranged between 0.8 and 1.8.

A fluorescein-lysine conjugate (FITC-Lys) was prepared by coupling 1 ml of 0.2 M L-Lysine with 0.4 mg of FITC. After 18 h of reaction at 4°C, most of the L-Lysine appeared coupled as resulted from the reduction of absorption at 492 $\mu$m, the maximum absorption peak of FITC. No further treatment of the conjugate was done. When used as a control reagent in incubation experiments, the FITC-Lys preparation was adjusted by dilution with PBS to the same concentration in fluorescein (measured at 492 $\mu$m) as the FITC-AFP derivative.

*Tritiated AFP*

Tritium radiolabelling of rat AFP was carried out using N-succinimidyl (2.3 [$^3$H])-propionate ([$^3$H]-NSP) (Amersham, England) and the procedure described by Kummer *et al.* (1981) for tritiation of monoclonal antibodies. Briefly 2 mg of lyophilized AFP dissolved in 1 ml of 0.1 M Na borate buffer pH 8.5, were added to 1 mCi of dried [$^3$H]NSP. The mixture was kept for 36 min at 4°C with stirring. Labelled AFP was separated from unincorporated reactants by Sephadex G-25 chromatography using PBS, pH 7.2, as the eluant. Fractions containing AFP were pooled. Aliquots, 50 $\mu$l each, were put in small vials and stored at −18°C until use. The specific activity of the preparation was of 29 $\mu$Ci mg$^{-1}$ AFP.

*AFP incubation of cultured cells*

Before incubation of the cultures with exogenous AFP, the medium in the dishes was replaced with fresh MEM solution supplemented with 5% NBCS and 100–150 $\mu$g ml$^{-1}$ of AFP, either as pure AFP or as FITC-AFP conjugate. After incubation under varied conditions of time, temperature, etc. (see '**Results**') the cultures were washed 3 times in cold PBS, fixed for 30 min. in cold ethanol-acetic acid (99:1; v/v) and dried in air. Dishes incubated with FITC-AFP were mounted in glycerol-PBS under a glass coverslip and viewed under epifluorescent illumination using a Leitz microscope. Pictures were taken if necessary. Treatment of cultures with proteins other than AFP was carried out in the same manner. Dishes incubated with AFP, FITC-AFP and controls were finally processed for immunocytochemical labelling (see below).

*High-resolution autoradiography*

Cloned cell lines were incubated in 1 ml fresh MEM medium to whom either 0.45 or 0.90 $\mu$Ci of [$^3$H]-AFP were added. After 3 h at 37°C, the cells were washed 3 times with PBS and fixed for 1 h at 4°C with 1.6% glutaraldehyde in 0.1 Sörensen phosphate buffer, pH 7. After several washes in buffer, including overnight, the cells were postfixed in 2% $OsO_4$ in the same buffer, for 1 h, at room temperature, scraped from the dishes and pelleted. The pellets were dehydrated in alcohol and embedded in Epon. Ultrathin sections were harvested on forward-coated copper grids and covered with a monogranular layer of Ilford L4 emulsion, using a loop. Autoradiograms were developed in a phenidon-containing developer, after gold latensification (Bouteille, 1976). The sections were finally stained with uranyl acetate and lead citrate.

*Immunochemical and immunocytochemical reactions*

Specific rabbit antisera to rat and mouse AFP were obtained as previously described (De Nechaud & Uriel, 1971; Hassoux *et al.*, 1977). Pure antibodies were isolated from their respective antisera by affinity chromatography on AFP-immuno-absorbents prepared by the procedure of Avrameas

& Ternynck (1969). Goat anti-rabbit IgG conjugated with peroxidase was from the Institut Pasteur (Paris). AFP localization in striated muscle sections or in cultured cell dishes was made by indirect immunoperoxidase technique with the appropriate controls, as described elsewhere (Trojan & Uriel, 1980).

### Results and discussion

### *AFP in immature striated muscle*

Immunoperoxidase staining for AFP was positive throughout the foetal period, as well as in neonate rats up to 8–10 days of age. Maximum staining, in both intensity and extension, was observed at the end of gestation. The labelling then declined progressively to total extinction 2 weeks after birth. At the cellular level (Figure 1) the localization of AFP was intracytoplasmic, and nuclei always appeared negative. The reaction was strong and clearly distinct along myofibrillar structures, as could be seen in both longitudinal and transversal sections of immature striated muscle.

Recent work from our laboratory has shown that several tissues, including striated muscle, of developing rats selectively accumulate radiolabelled AFP when the protein is injected into pregnant rats or neonates (Villacampa *et al.*, submitted). This confirms the conclusion reported above (see **Introduction**) that the high content of AFP in striated muscle results from active incorporation of the protein.

### *AFP uptake by cloned cell lines from rhabdomyosarcoma*

Culture dishes of the parental line Rh 9–4–0 and of the 2 cloned cell lines, F 9–4 and J 9–4, were grown for 48 h as described in **Materials and methods**. Then, they were incubated at 37°C in air-$CO_2$ humidified atmosphere for either 4, 8 or 16 h in MEM-NBCS medium complemented with 150 $\mu g\,ml^{-1}$ of rat or mouse AFP. Controls consisted of dishes incubated for the same periods of time in MEM-NBCS medium alone. After washing and fixation, the dishes were processed for immunocytochemical localization of AFP with homologous antibodies. The presence of intracellular AFP could be demonstrated in all dishes incubated with the protein. Control dishes were negative. AFP labelling was also negative in cultures incubated or not with AFP, but subsequently treated with rabbit normal IgG instead of antibodies to AFP. No significant differences were observed after AFP treatment for 4, 8 or 16 h, nor between cultures incubated with either rat or mouse AFP.

Two representative examples of strong AFP uptake by cultured cell of rhabdomyosarcoma—the parental line and clone F 9–4/22—are shown in Figure 2. The labelling in both was intracytoplasmic and extended to straight processes and filaments interconnecting cells. A particularly dense labelling was seen in large elements with fused nuclei and myotubular-like morphology.

Preliminary autoradiograms at the electron microscope level of clone F 9–4/22 incubated with [$^3$H]-AFP are shown in Figure 3. Silver grains were associated with coated pits and occasionally with dictyosomes. A few autoradiographic grains were localized over cytoplasmic regions containing ribosomes and dilated ergastoplasmic cisternae. Most silver grains however were concentrated over lipid droplets, often grouped in the cell cytoplasm, a localization which, to our knowledge, has never been reported for proteins or peptide hormones internalized by receptor-mediated endocytosis. The number of silver grains observed over the cells was significantly higher when the cultures were incubated in the presence of 0.90 instead of 0.45 $\mu$Ci [$^3$H]-AFP $ml^{-1}$. There was hardly any silver grain background outside the cells or in the cells from monolayers untreated with [$^3$H]-AFP.

To better explore the possible dependence time of AFP-uptake, cultures of clone J 9–4/2 were treated at 37°C with 150 $\mu g\,ml^{-1}$ of the fluorescent conjugate FITC-AFP (rat). The reaction was arrested at variable periods of time and the fluorescence viewed under microscopic examination. Slight but distinct labelling was observed as early as 15 min after incubation. Small fluorescent patches appeared on the cell membrane and along filaments interconnecting cells. Fluorescence increased with incubation time and reached the whole cytoplasm. (Figure 4). To demonstrate that the observed labelling was due to the FITC-protein conjugate and not to free fluorescein, dishes were post-incubated with antibodies to rat AFP as the first step in the immunocytochemical localization of the protein (for details see **Materials and methods**). As illustrated in Figure 4 the patterns of fluorescence and of immunoperoxidase staining were similar.

The specificity of AFP uptake was assessed by comparing the internalization of a series of fluorescent conjugates of AFP and of other proteins. After incubation for 3–4 h at 37°C, the fluorescence patterns of rat AFP and serum albumin were analogous. On the contrary, no intracellular fluorescence could be observed in cultures treated in the same conditions with FITC-egg albumin. The control conjugate FITC-Lys gave also negative results. On the other hand, a clear

**Figure 1** Transverse sections of the tongue of a 19-day rat foetus. Immunoperoxidase-staining with rabbit anti-rat AFP antibodies. Note the strong positive labeling of myofibrillar structures in both (a) and (b) pictures. Nuclei (arrows) appear unstained. ×400.

**Figure 2** Cultures (72 h) of the rhabdomyosarcoma parental cell line, Rh-9-4/0, and clone F 9-4/22 incubated at 37°C for 4 h in MEM-NBCS medium containing 150 $\mu g\,ml^{-1}$ of rat AFP. Immunoperoxidase staining. Note positive intracytoplasmic labelling of spindle-like cells (a) and (b) and of large elements with myotubular-like structure (b). ×400.

species-specificity resulted when several AFP from different origin were comparatively assayed. Thus, while similar patterns were associated with rat and mouse AFP, two strong immunochemically cross-reacting proteins, the labelling with pig AFP was much weaker and it was negative with chicken AFP. Whatever the protein assayed, all positive fluorescence patterns at 37°C vanished to near completion when the cultures were incubated at 0°C.

The interaction of cloned cell line J 9–4/10 deserves special attention. When incubated with FITC-AFP (150 $\mu g\,ml^{-1}$ at 37°C), fluorescence appeared mostly limited to the cell membrane, and the same pattern was observed when AFP was visualized by the indirect immunoperoxidase technique (Figure 5). This suggests that clone J 9–4/10, while keeping the ability to bind AFP, and contrary to its parental cell line, had lost the property of AFP internalization. Such behaviour was unique among the clones examined.

Previous work with primary cultures of foetal brain cells has demonstrated that the ability to incorporate AFP is not displayed by undifferentiated precursors, but seems restricted to elements with phenotypic characteristics of maturing neurons (Uriel *et al.*, 1981). In this regard we report that only clone J 9–4/10 lacks the property of AFP internalization. It is also interesting to note that this clone has morphological characteristics which differ from all others examined, due to the absence of myotubular-like structures and spindle-like cells, and a predominance of round or oval, poorly-differentiated elements. Whether such behaviour may be ascribed to its degree of differentiation, or whether it results from some defect in the mechanism of protein uptake of this cell line requires further investigation.

A great variety of molecules, including serum proteins, which bind to specific receptors on the cell membrane are subsequently internalized by a

**Figure 3** Rhabdomyosarcoma cells were incubated at 37°C in the presence of [$^3$H]-AFP (0.90 $\mu$Ci) for 3.5 h. Autoradiographs were developed after 7.5 months exposure. (a): four silver grains are localized over the Golgi region, on the right side, and the other ones over lipid droplets, on the left side. × 33000. (b): two silver grains are localized near a coated pit (arrow). × 33000. (c): silver grains are concentrated over grouped lipid droplets, near the nucleus (N). × 24000. (d): silver grains are concentrated over two lipid droplets; two other ones are localized over an ergastoplasmic cisterna, on the right side. × 39000.

**Figure 4** Culture (48 h) of clone J 9-4/22: (a) Incubated at 37°C for 6 h in MEM-NBCS medium additioned with $150 \mu g ml^{-1}$ rat AFP-FITC. Intracellular uptake of AFP viewed under epifluorescent illumination. (b) Immunoperoxidase staining of AFP after post-incubation of the culture with antibodies to AFP. ×400.

mechanism called receptor-mediated endocytosis (see reviews by Goldstein *et al.*, 1979 and Besterman & Low, 1983). The morphological data reported above suggest that the same mechanism may underlie the incorporation of AFP by rhabdomyosarcoma cultured cell lines. The temperature dependence and the relative high degree of species-specificity associated with AFP uptake, as well as the ultrastructural autoradiographs of internalized AFP, seem to support such an hypothesis. It is also well known that some proteins that enter cells through receptor-mediated endocytosis are not degraded but instead are directed to specific subcellular organelles (Goldstein *et al.*, 1979). The selective accumulation into lipid droplets of [$^3$H]-AFP conforms to that particular behaviour. On the other hand, the incorporation of rat serum albumin by rhabdomyosarcoma cells is consistent with previous observations showing that the intracellular presence of serum albumin in the immature central nervous system and other tissues of developing animals follows the same pattern of cell and tissue distribution than AFP (Mollgard *et al.*, 1979; Toran-Allerand, 1980; Trojan & Uriel, 1979, 1982).

Within the past years, numerous *in vitro* cell systems have been described in which non-phagocytic cells use endocytosis to internalize proteins. Work in progress in our laboratory is showing that cell types other than rhabdomyosarcoma and noteworthy neuroblastoma cells, adult and foetal fibroblasts, may incorporate AFP. The interest of the present study lies, partially, in the reappearance in cloned cell lines derived from a primary rhabdomyosarcoma of a property pertaining to immature striated muscle.

**Figure 5** Culture (48 h) of clone J 9-4/10 treated for 4 h at 37°C with AFP-FITC (120 $\mu g\,ml^{-1}$) in MEM-NBCS medium. AFP localisation appears restricted to cell membranes and stright processes. The protein is visualized by direct fluorescence examination in (a) and by the immunoperoxidase technique, after post-incubation with antibodies to AFP in (b).

The latter conforms with a large body of information obtained in the past on the resurgence of foetal patterns of gene expression in cancer (oncofoetal antigens, isoenzymes, etc). (Ibsen & Fishman, 1979; Weinhouse, 1982; Uriel, 1979). In the present case the resumed phenotypic trait probably implies the expression of specific AFP receptors and the reactivation of a mechanism of receptor-mediated endocytosis of this protein operational in muscle cells only during ontogenesis.

The present work has been supported, in part, by a grant of the Fundation pour la Recherche Médicale Française.
M. Geuskens is senior research associate of the National Fund for Scientific Research (Belgium).

**References**

ABE, T., KOMATSU, M., TAKEISHI, M. & TSUNEKANE, T. (1976). The alphafetoprotein level in the sera of bovine fetuses and calves. *Jap. J. Vet. Sci.*, **38**, 339.

AVRAMEAS, S. & TERNYNCK, T. (1969). The cross-linking of protein with glutaraldehyde and its use for the preparation of immunoadsorbents. **6**, 53.

BASTERIS, B. (1979). Immunofluorescent localization of alphafetoprotein and albumin in embryonic fetal and newborn rat. In *Carcinoembryonic Proteins* (Ed. Lehman) Elsevier North Holland: Biomed. Press; II p. 353.

BESTERMAN, J.M. & LOW, R.B. (1983). Endocytosis: a review of mechanisms and plasma membrane dynamics. *Biochem. J.* **210,** 1.

BOUTEILLE, M. (1976). The "LIGOP" method for routine ultrastructural autoradiography. A combination of single grid coating, gold latensification and phenidon development. *J. Microscopie Biol. Cell,* **27,** 121.

DZIADEK, M. & ADAMSON, E. (1978). Localization and synthesis of alphafoetoprotein in postimplantation mouse embryos. *J. Embryol. Exp. Morph.,* **41,** 182.

GOLDSTEIN, J.L., ANDERSON, R.G.W. & BROWN, M.S. (1979). Coated pits, coated vesicles and receptor-mediated endocytosis. *Nature,* **279,** 679.

HASSOUX, R., BERGES, J. & URIEL, J. (1977). Affinity chromatography of mouse alphafoetoprotein (AFP) on oestradiol-Sepharose absorbants. *J. Steroid Biochem.,* **8,** 127.

IBSEN, K.H. & FISHMAN, W.M. (1979). Developmental gene expression in cancer. *Biochim. Biophys. Acta.,* **566,** 243.

KUMMER, U., THIEL, U., DOXIADIS, I., EULITZ, M., SLADOLJEV, S. & THIEL-FELDER, S. (1981). Tritium radiolabeling of antibodies to high specific activity with N-succinimidyl (2,3-$^{3}$H) propionate: use in detecting monoclonal antibodies. *J. Immunol. Methods,* **42,** 367.

LAMPREAVE, F., CALVO, M., NAVAL, J. & PINEIRO, A. (1982). Long-chain fatty acids bound to 2 alpha fetoprotein and to serumalbumin from fetal and adult pig. *Comp. Biochem. Physiol.,* **73B,** 823.

MOLLGÄRD, K., JACOBSEN, M., KRAG JACOBSEN, G., PRETORIUS CLAUSEN, P. & SAUNDERS, N.R. (1979). Immunohistochemical evidence for intracellular localization of plasma proteins in human foetal choroid plexus and brain. *Neurosci, Letters,* **14,** 85.

MORO, R. & URIEL, J. (1981). Early localization of alpha-foetoprotein in the developing nervous system of the chicken. *Oncodevel. Biol. Med.,* **2,** 391.

DE NECHAUD, B. & URIEL, J. (1971). Antigèns cellulaires transitoires du foie de rat. I. Secrétion et synthèse des protéines sériques foeto-specifiques au cours du développement et de la régénération hépatique. *Int. J. Cancer,* **8,** 71.

SWEENEY, F., POT-DEPRUN, J., POUPON, M.F. & CHOUROULINKOV, I. (1982). Heterogeneity of growth and metastatic behavior of cloned cell lines derived from a primary rhabdomyosarcoma. *Cancer Res.,* **42,** 3776.

TORAN-ALLERAN, C.D. (1980). Coexistence of $\alpha$-fetoprotein, albumin and transferrin immunoreactivity in neurons of the developing mouse brain. *Nature,* **286,** 733.

TROJAN, J. & URIEL, J. (1979). Localisation intracellulaire de l'alphafoetoprotein et de la sérumalbumine dans le système nerveux central du rat au cours du développement foetal et postnatal. *C.R. Acad. Sci.,* (Paris) **289D,** 1157.

TROJAN, J. & URIEL, J. (1980). Immunocytochemical localization of alphafoetoprotein in the developing rat brain. *Oncodevel. Biol. Med.,* **1,** 107.

TROJAN, J. & URIEL, J. (1982). Immunocytochemical localization of alphafoetoprotein (AFP) and serumalbumine (Alb) in ecto-, meso- and endodermal tissue derivatives. *Oncodevel. Biol. Med.,* **3,** 13.

URIEL, J. (1979). Retrodifferentiation and the fetal patterns of gene expression in cancer. *Ad. Cancer Res.,* **29,** 127.

URIEL, J., FAIVRE-BAUMAN, A., TROJAN, J. & FOIRET, D. (1981). Immunocytochemical demonstration of alphafoetoprotein uptake by primary cultures of foetal hemisphere cells from mouse brain. *Neurosci. Letters,* **27,** 171.

URIEL, J., TROJAN, J., DUBOUCH, P. & PINEIRO, A. (1982). Intracellular alphafoetoprotein and albumin in the developing nervous system of the baboon. *Pathol. Biol.,* **30,** 79.

VILLACAMPA, M.J., LAMPREAVE, F., CALVO, M., PINEIRO, A. & URIEL, J. (1983). Incorporation of radiolabeled alphafetoprotein in the brain and other tissues of the developing rat (submitted to Develop. Brain Res.).

WEINHOUSE, S. (1982). What are isozymes telling us about cancer? *J. Natl Cancer Inst.,* **68,** 343.